# Characterization of the rainbow trout transcriptome using Sanger and 454-pyrosequencing approaches

**DOI:** 10.1186/1471-2164-11-564

**Published:** 2010-10-13

**Authors:** Mohamed Salem, Caird E Rexroad, Jiannan Wang, Gary H Thorgaard, Jianbo Yao

**Affiliations:** 1 Laboratory of Animal Biotechnology and Genomics, Division of Animal and Nutritional Sciences, West Virginia University, Morgantown, WV 26506, USA; 2 National Center for Cool and Cold Water Aquaculture, Kearneysville, WV 25430, USA; 3 School of Biological Sciences, Washington State University, Pullman, WA 99164, USA

## Abstract

**Background:**

Rainbow trout are important fish for aquaculture and recreational fisheries and serves as a model species for research investigations associated with carcinogenesis, comparative immunology, toxicology and evolutionary biology. However, to date there is no genome reference sequence to facilitate the development of molecular technologies that utilize high-throughput characterizations of gene expression and genetic variation. Alternatively, transcriptome sequencing is a rapid and efficient means for gene discovery and genetic marker development. Although a large number (258,973) of EST sequences are publicly available, the nature of rainbow trout duplicated genome hinders assembly and complicates annotation.

**Results:**

High-throughput deep sequencing of the Swanson rainbow trout doubled-haploid transcriptome using 454-pyrosequencing technology yielded ~1.3 million reads with an average length of 344 bp, a total of 447 million bases. *De novo *assembly of the sequences yielded 151,847 Tentative Consensus (TC) sequences (average length of 662 bp) and 224,391 singletons. A combination assembly of both the 454-pyrosequencing ESTs and the pre-existing sequences resulted in 161,818 TCs (average length of 758 bp) and 261,071 singletons. Gene Ontology analysis of the combination assembly showed high similarities to transcriptomes of other fish species with known genome sequences.

**Conclusion:**

The 454 library significantly increased the suite of ESTs available for rainbow trout, allowing improved assembly and annotation of the transcriptome. Furthermore, the 454 sequencing enables functional genome research in rainbow trout, providing a wealth of sequence data to serve as a reference transcriptome for future studies including identification of paralogous sequences and/or allelic variation, digital gene expression and proteomic research.

## Background

Rainbow trout (*Oncorhynchus mykiss*) are widely distributed fish species cultured in regions with cold and cool water temperatures around the world. They are important food and sport fish and the most cultivated cold freshwater fish in the US [1-3]. In addition, rainbow trout is one of the most rigorously studied fishes in many research areas including carcinogenesis, toxicology, comparative immunology, disease ecology, physiology and nutrition. For over a century, rainbow trout has been used as a model research species; more is known about the biology of rainbow trout than any other fish species. Rainbow trout is a member of the Salmonidae family. During the last decade more than 26,000 scientific papers have been published on salmonids. Rainbow trout can also serve as a surrogate for research needed on other economically important salmonid species such as Atlantic and Pacific salmon and char species [4]. This wealth of knowledge and interest in rainbow trout has guided increased attention to developing and using genomics tools, as evidenced by the considerable accumulation of genomic resources in recent years from several groups around the world [5,6]. However, the rainbow trout genome sequence is not available yet, and this lack of information hinders the contemporary approaches to identification/characterization of important genes and genetic markers for aquaculture applications and biomedical research. Until the reference genome sequence becomes available, transcriptome sequencing is a fast and efficient means for gene discovery and genetic marker development. Transcriptome sequencing enables functional genomics studies including global gene expression, single nucleotide polymorphism (SNP) discovery, quantitative trait loci analysis and genome scans of diversity [7]. Rainbow trout is a significant fish model in terms of expressed sequence tags (ESTs) [8]. International large-scale EST sequencing projects for rainbow trout have generated large number of ESTs (258,973) that are publicly available through the Rainbow Trout Gene Index database [8-10].

As a member of the family Salmonidae, rainbow trout has descended from a tetraploid ancestor[11]. A whole genome duplication event occurred 25-100 mya and the genome is estimated to be 1/3 of the way toward re-diploidization [11,12]. About 50% of protein-coding loci examined in salmonid species show duplicate gene expression [13]. The nature of duplicated rainbow trout genome hinders assembly and complicates annotation of the EST sequences as well as identification of SNPs [14]. Attempts to assemble and annotate the rainbow trout ESTs yielded 40,320 Tentative Consensus (TC) sequences, 49,408 singletons and 26,861 UniGenes [15,16]. Previous efforts aimed at SNP discovery for rainbow trout using ESTs were unsuccessful, mainly, due to difficulties parsing allelic variation from the high frequency of duplicated genes [14]. To better characterize the rainbow trout transcriptome and improve discovery of allelic variations, a haploid transcriptome sequence is needed. Doubled haploid clonal lines, fish that have two identical copies of each chromosome, were established in salmonids, using androgenesis and gynogenesis; whole chromosome set manipulation techniques [17,18]. In androgenesis, eggs are gamma-irradiated to destroy DNA and then fertilized with untreated sperm. Then, a temperature or pressure shock is used to interrupt mitosis, resulting in completely homozygous doubled haploid fish. Similarly, in gynogenesis, sperm are treated with ultraviolet radiation to induce development of untreated eggs before the temperature or pressure shock treatment. To create a clonal line, completely homozygous and identical doubled haploid, population, a single homozygous fish is reproduced through a second generation of chromosome set manipulation [17,18].

Recently, 454-pyrosequencing has become relatively rapid and cost-effective method for sequencing with progressively increasing depth and coverage. Therefore, it is a very suitable technique for *de novo *transcriptome sequencing for non-model species such as rainbow trout [7,19-21]. The objectives of this study were to: 1) sequence and characterize the transcriptome of an individual from the Swanson doubled haploid rainbow trout clonal line using 454-pyrosequencing to produce a transcriptome reference sequence for identification of gene duplications, distinguishing true/false SNPs and future genomic/transcriptomic sequencing; and 2) use the 454-pyrosequencing and the Sanger-based EST data to characterize the rainbow trout transcriptome with improved assembly and annotation for digital gene expression and proteomic research in the future.

## Results and Discussion

### Sanger-based sequencing of the rainbow trout transcriptome

In the last few years, large-scale EST sequencing projects for rainbow trout have been undertaken in several laboratories worldwide. Rise and coworkers reported 14,544 ESTs (mostly 3'-sequences) from 6 rainbow trout cDNA libraries [10]. Govoroun and coworkers reported a total of 96,472 ESTs sequenced from three normalized and subtracted cDNA libraries including one highly complex pooled-tissue library from 14 different tissues [8]. A substantial number of ESTs were generated in our labs, currently representing 47% of the total number of available rainbow trout sequences (124,138 out of 258,973) (Rainbow Trout Gene Index database release 7.0) [9,15]. Over 87,000 of these ESTs were sequenced from a normalized library constructed from mRNA pooled from several tissues including; muscle, brain, gill, spleen, liver, kidney and juvenile tissues [9,10]. The normalization and pooling strategies were used to increase probability of identifying unique and diverse set transcripts. Clones were randomly picked and sequenced until library sequencing is saturated; low probability of discovering a new mRNA reached due to the redundancy effect. Collectively, the international efforts of sequencing the rainbow trout transcriptome have thus far generated 290,735 ESTs that are publicly available through the GenBank [22]. Table 1 shows summary statistics of the major rainbow trout EST projects listed in the Rainbow Trout Gene Index database [9,15]. In this database, all EST sequence data were integrated and assembled producing 90,019 total unique sequences [8-10]. A total of 209,565 ESTs (81%) were clustered in 40,320 TCs and 49,408 ESTs (19%) were singletons. There is an average of 5.2 EST per TC (Rainbow Trout Gene Index database release 7.0) [23]. Most of the sequences were derived from 5'- or 3'-sequencing and thus are less likely to cover coding regions, which hinders the EST annotations. Table 2 shows summary statistics of the Sanger-based ESTs assembly compared to the below-mentioned *de novo *assembly of the 454-pyrosequencing and a combination assembly of both datasets.

**Table 1:** Summary statistics of the major rainbow trout Sanger-based EST projects

Library Name	Supplier	Tissue/Organ	# in TCs	# of singletons	Total ESTs
NCCCWA 1RT	NCCCWA-USDA	Pooled	34927	11452	46379
NCCCWA 02RT	NCCCWA-USDA	Pooled	14506	3452	17958
NCCCWA 03RT	NCCCWA-USDA	Pooled juvenile	17412	651	18063
NCCCWA 04RT	NCCCWA-USDA	Pooled juvenile	2198	407	2605
NCCCWA 05RT	NCCCWA-USDA	Pooled juvenile	1789	282	2071
NCCCWA 07RT	NCCCWA-USDA	Pituitary	3208	321	3529
NCCCWA 09RT	NCCCWA-USDA	Testis	4615	537	5152
NCCCWA 10RT#1-4	NCCCWA-USDA	Oocyte	254	78	332
NCCCWA 10RT#2	NCCCWA-USDA	Oocyte	147	19	166
NCCCWA 10RT#3	NCCCWA-USDA	Oocyte	14594	3060	17654
NCCCWA 10RT#4	NCCCWA-USDA	Oocyte	68	16	84
leuk	NCCCWA-USDA	Anterior kidney/spleen	6223	166	6389
EMB	NCCCWA-USDA	Embryo	3529	227	3756
AGENAE Rainbow trout multi-tissues library (tcaa)	INRA - SCRIBE	Adipose, blood, brain, gonad	863	256	1119
AGENAE Rainbow trout normalized testis library (tcab)	INRA - SCRIBE	Testis	730	294	1024
AGENAE Rainbow trout multi-tissues subtracted library (tcay)	INRA - SCRIBE	Adipose, blood, brain, gonad	17859	8433	26292
AGENAE Rainbow trout normalized ovarian library (tcby)	INRA - SCRIBE	Ovary	4714	204	4918
AGENAE Rainbow trout normalized multi-tissues library (tcac)	INRA - SCRIBE	Adipose, blood, brain, gonad	3265	713	3978
AGENAE Rainbow trout subtracted multi-tissues library (tcav)	INRA - SCRIBE	Adipose, blood, brain, gonad	2516	850	3366
Agenae (tcbj)	INRA - SCRIBE		179	17	196
AGENAE Rainbow trout normalized multi-tissues library (tcad)	INRA - SCRIBE	Adipose, blood, brain, gonad	5015	1131	6146
AGENAE Rainbow trout normalized testis library (tcbi)	INRA - SCRIBE	Testis	11494	3603	15097
AGENAE Rainbow trout multi-tissues-normalized (tcbk)	INRA - SCRIBE	Multi-tissues	21132	5757	26889
AGENAE Rainbow trout multi-tissues library (tcce)	INRA - SCRIBE	Embryos to adults	6211	1664	7875
Oncorhynchus mykiss reproductive	University of Victoria	Gonads	5387	848	6235
Oncorhynchus mykiss Chilliwack River steelhead whole	University of Victoria	Whole embryo/juvenil	3760	246	4006
Oncorhynchus mykiss Tzenaicut Lake whole	University of Victoria	Whole embryo/juvenil	2266	188	2454

Data from the National Center for Cool and Cold Water Aquaculture (NCCCWA-USDA) and the National Institute for Agricultural Research (INRA) constitute 47% and 37%, respectively.

**Table 2:** Summary statistics of the Sanger-based assembly, 454-pyrosequencing assembly and a combination assembly of both datasets

	Combination assembly	Sanger assembly	454 assembly
**Total sequences**	521 M bases	74 M bases	447 M bases
**Total no. of ESTs**	1,380,311	258,973	1,290,292
**No. of unique sequences**	422,889	90,019	376,238
**No. of sequences assembled**	1,119,240 (81%, Ave = 394 bp)	209,565 (81%)	1,065,901 (83%, Ave = 374 bp)
**No. of sequences unassembled**	261,071 (Ave = 417 bp)	49,408	224,391 (Ave = 345 bp)
**Total no. of contigs**	161,818 (Ave = 758 bp)	40,320 (Ave = 999 bp)	151,847(Ave = 662 bp)
**Total no. of large contigs (>500 bp)**	101,464	78,572	84,810

### 454-Pyrosequencing of the rainbow trout transcriptome

A 454-pyrosequencing library was constructed from 14 tissues collected from a single immature male homozygous doubled-haploid fish from the Swanson clonal line, the same line used for BAC library construction (12). The 454 library was titrated in three one-sixteenth-plate regions to determine the optimal bead library concentration. After titration, the library was sequenced in two half-plate regions of a PicoTiterPlate. Great wealth of sequence data were obtained from the pyrosequencing runs, yielding six times more sequence data than all the currently available Sanger-based data. A total of 1,416,404 ESTs (average length 344 bp) and 447 million bases were obtained from 454-pyrosequencing. The primary sequence data were deposited in GenBank Short Read Archive (SRA) under accession number SRA009276.8. Of these, 1,290,292 (91%) passed quality standards (primer filtering; sequences that contain homopolymers and shorter than 100 bp were filtered out).

**Figure 1:**
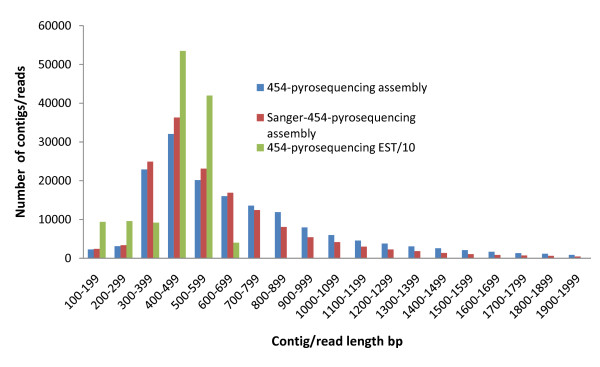
**Average length distribution of assemblies and pyrosequencing reads of the rainbow trout ESTs**. Average lengths of the combination assembly contigs are more than those of the 454-pyrosequencing assembly over the 100-700 bp length range, indicating that addition of the Sanger-based data improved assembly of the short 454-pyrosequenceing reads up to 700 bp. However, over the 700-2000 bp length range, average contig lengths of the combination assembly are less than those of the 454-pyrosequencing assembly indicating that contigs longer than 700 bp of the combination assembly were mainly derived from the 454-pyroseqeuncing data, possibly because most of the 454-pyrosequencing reads were 400-600 bp. Number of the 454-pyrosequencing reads is divided by 10 for scaling.

Sequences that passed quality standards were clustered and *de novo *assembled. A total of 1,065,901 reads were integrated into 151,847 TCs leaving 224,391 singletons; yielding a total of 376,238 unique sequences (Table 2). The *de novo *assembly sequence data were deposited in the Transcriptome Shotgun Assembly (TSA) database with the accession numbers EZ763335-EZ915181. The percentage of the assembled 454-pyrosequencing sequence reads was 83% compared to 81% of the Sanger-based EST data. The average length of the 454-pyrosequencing TCs was 662 bp, compared to 999 bp of the Sanger-based data. Distribution of 454-assembly showed a wide range of contig lengths with most contigs ranging between 300 and 900 bp (Figure 1). The number of contigs larger than 500 bp was 84,810 (56%). *De novo *assembly of pyrosequencing transcriptomic data from other non-model species yielded 25-88% ESTs assembled into contigs of average length 197-233 bp [7,24,25]. Successful assembly of rainbow trout pyrosequencing data into relatively long contigs is apparently due to the length of the individual reads and/or thorough coverage of the transcriptome.

The 454-pyrosequencing data were generated and assembled from a doubled-haploid rainbow trout clonal line to address problems associated with the nature of the rainbow trout duplicated genome that hinders assembly and complicates annotation [11,14]. The 454-pyrosequencing data were assembled to serve as a reference sequence for future studies to identify gene duplications from allelic variations and to distinguish true/false SNPs. Sequence data were *de novo *assembled using the default parameters of the SeqMan NGen software (see Materials and Methods for details).

The 454-pyrosequencing library identified large number of new genes. A total of 282,357 gene transcripts were identified by subtracting the Sanger-based sequences from the 454-pyrosequencing sequence data. The subtraction was done by using BLASTn with an e-value below 1e-10, an alignment longer than 99 bp and 95% sequence identity within the aligned region (data not shown).

A combination of both the 454-pyrosequencing ESTs and the Sanger-based sequences offered the maximum amount of genetic information available for rainbow trout so far; 521 M bp, 1,380,311 ESTs and 422,889 unique sequences (Table 2). The combination assembly sequence data are available at the NAGRP Aquaculture Genome Projects [26]. With estimates of 2.4 to 3.0 × 10^9 ^bp genome size of the rainbow trout and 1% of the genome being mRNAs, the combined data provide an estimated 19× depth of coverage of the rainbow trout transcriptome [27,28]. The combination assembly increased the number of TCs to 161,818 (average contig length of 758 bp) leaving 261,071 singletons. A comparison of the percentage of the assembled reads in the combination assembly (81%) to the 454-pyrosequencing and the Sanger-based assemblies (83% and 81%, respectively) indicates that combining the two sequence data did not improve the assembly percentage of each individual sequence dataset (Table 2).

Figure 1 shows contig length distribution of the two assemblies compared to 454-pyrosequence average read length distribution. The figure illustrates that over 100-700 bp length range, average contig lengths of the combination assembly are more than that of the 454-pyrosequencing assembly, indicating that adding the Sanger-based data improved assembly of the short 454-pyrosequences reads up to 700 bp. However, over 700-2000 bp length range, average contig lengths of the combination assembly are less than that of the 454-pyrosequencing assembly indicating that contigs longer than 700 bp of the combination assembly were mainly derived from the 454-pyroseqeuncing data, possibly because most of the 454-pyrosequencing reads were 400-600 bp. The relatively short read lengths of the 454 pyrosequencing compared to Sanger sequencing is a major concern of the 454-pyrosequencing [25,29]. The 454/GS-Titanium sequencing platform, used in this study, has been used to address this issue and lead to successful assembly of the 454 pyrosequencing data, which make this technology suitable for *de novo *transcriptome sequencing of non-model species such as aquaculture species [7,30].

### Functional annotation and Gene Ontology analyses

**Figure 2:**
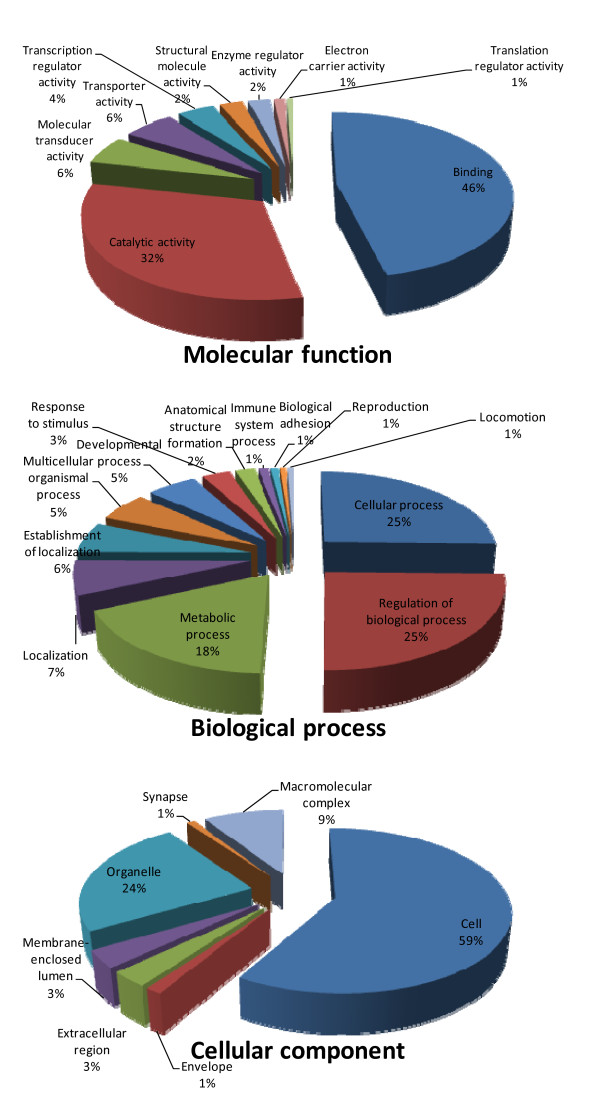
**Gene Ontology (GO) assignment (2^nd ^level GO terms) of the rainbow trout Sanger-based and 454-pyrosequncing combination assembly**. Biological processes constitute majority of GO assignment of the transcripts (17,694 counts, 39%), followed by cellular components (16,452 counts, 36%) and molecular function (11,160 counts, 25%).

Functional annotation of transcripts of the combination assembly was carried out by BLASTx search against the NCBI non-redundant protein database using the Blast2GO suite [31]. BLAST result accessions were used to retrieve associated gene names and Gene ontology (GO) terms. Gene ontology is widely used to standardize representation of genes across species and provides a controlled vocabulary of terms for describing gene product [32]. Biological processes constituted the largest class of GO assignment of the transcripts (17,694 counts, 39%), followed by cellular components (16,452, 36%) and molecular function (11,160, 25%). Level 2 GO assignments within the three categories are summarized in Figure 2. Of the biological process categories, 25% of the genes are associated with cellular processes and 25% are related to regulation of biological process. Within the molecular functional category, 46% are related to protein binding followed by 32% catalytic activity. Of the cellular components, 59% of the genes are related to the cell and 24% were related to organelles.

**Figure 3:**
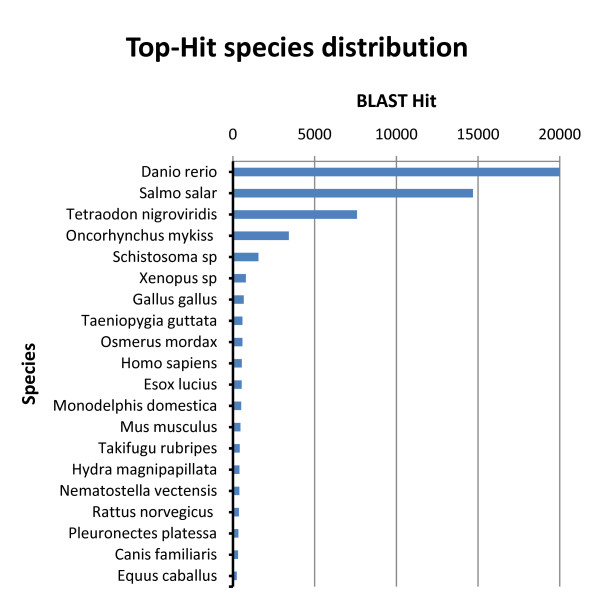
**BLASTx top-hit species distribution of gene annotations showing high homology to fish species with known genome sequences**. Only 5.7% of the BLAST hits matched rainbow trout protein sequences due to the limited number of the rainbow trout proteins (6,915) that are currently available in the NCBI database (compared to 141,396 proteins in Zebrafish).

A BLASTx top-hit species distribution of gene annotations showed highest homology to Zebrafish (*Danio rerio*), followed by Atlantic salmon (*Salmo salar*) and Puffer fish (*Tetraodon nigrovirdis*) (Figure 3). In addition, the trout sequences had significant homologies to four more fish species including Japanese pufferfish (*Takifugu rubripes*), rainbow smelt (*Osmerus mordax*), Northern pike (*Esox lucius*) and European plaice (*Pleuronectes platessa*). These results indicate a high level of phylogenetic conservation of the rainbow trout gene content compared to these fish species. On the other hand, the model fish species Medaka fish (*Oryzias latipes*) and Stickleback fish (*Gasterostreus aculeatus*) showed very low homology to the trout sequences. It is worth mentioning that only 5.7% of the BLASTx top-hits matched rainbow trout protein sequences. This may be explained on the basis of the limited number of the rainbow trout proteins (6,915) that are currently available in the NCBI database compared to other fish species with known genome sequences (141,396 proteins in the case of Zebrafish) and suggests identification of large number of new genes by the 454-pyrosequencing library [22]. The results of BLASTx top-hit of fish species are provided in Additional file 1, Table S1.

**Figure 4:**
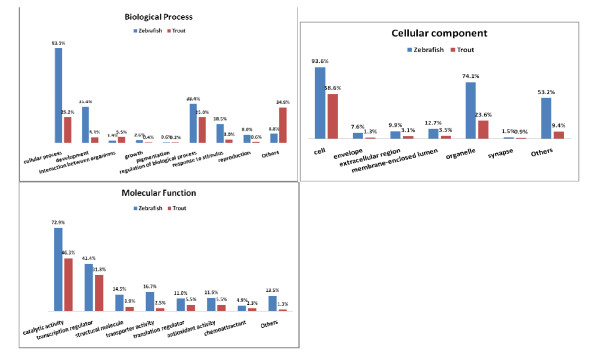
**Gene Ontology (2^nd ^level GO terms) comparison of the rainbow trout combination assembly showing high similarity to the transcriptome of Zebrafish (with known genome sequence)**.

Based on the BLASTx top-hit species distribution of gene annotations, Zebrafish was chosen for a detailed Gene Ontology comparison (Figure 4). Distribution profile and percentage of clustered 2^nd ^level Gene Ontology terms of the combination assembly showed high similarity to that of the Zebrafish suggesting an unbiased representation of the combination assembly to the rainbow trout transcriptome. Similarities of the rainbow trout and Zebrafish transcripts as indicated by distributions of clusters of different categories of the biological processes, cellular components and molecular functional implies that the combination assembly represents the rainbow trout tissue complexity and that normalization of the 454-pyrosequencing pooled-tissue library and the Sanger-based sequences were effective in reducing redundancy (Figure 4).

## Conclusion

A hybrid approach of the high throughput 454-pyrosequencing together with the traditional Sanger-based sequences was used to generate high-quality draft assemblies of the rainbow trout transcriptome. The 454-pyrosequencing data were generated and assembled from a Swanson doubled-haploid rainbow trout individual to address problems associated with the nature of the rainbow trout duplicated genome; to serves as a reference sequence for future studies to identify gene duplications from allelic variations and to distinguish true/false SNPs.

The 454-pyrosequencing yielded a great wealth of sequence data. Assembly of the 454-pyrosequencing data together with the Sanger-based EST sequencing data provides 19× depth of coverage of the rainbow trout transcriptome, allowing robust functional annotation and gene ontology designation of the rainbow trout transcripts, and providing a reference sequence for digital gene expression, proteomic studies and QTL mapping.

While the released assembly sequences significantly increased the suite of transcriptome sequences available for rainbow trout that enables functional genome research, next-generation sequence assemblies may contain incorrectly assembled contigs, special attention should be given to this fact.

## Methods

### Production of doubled haploid rainbow trout

The doubled haploid trout from the Swanson clonal line were produced at Washington State University (WSU) as described previously[17,18] Homozygous rainbow trout were produced by androgenesis and by gynogenesis by blockage of the first cleavage [33]. Fish were raised to sexual maturity in recirculating systems at WSU. Clones of homozygous individuals were produced by collecting sperm from homozygous males and doing another cycle of androgenesis as described above, or by stripping the eggs from homozygous androgenetically or gynogenetically produced females and performing gynogenesis by retention of the second polar body [34]. The eggs were incubated in Heath trays at 12°C until hatching, then transferred to re-circulating systems for grow out.

### Tissue collection and RNA isolation

Tissues were collected from a single immature (~2-year-old, ~250 g) male homozygous doubled-haploid fish from the Swanson River clonal line[18]. Tissues were flash frozen in liquid nitrogen and shipped on dry ice from WSU to WVU then stored at -80°C until total RNA isolation. Total RNA was isolated using TRIzol™ (Invitrogen, Carlsbad, CA) from 14 tissues including white muscle, red muscle, testis, spleen, kidney, head kidney, pituitary, stomach, brain, heart, intestine, gill, skin and liver. Equal masses of total RNAs from various tissues were pooled and used to construct the 454-pyrosequencing library.

### 454-cDNA Library construction

Library preparation and 454-pyrosequencing and sequence assemblies were done at Roy J. Carver Biotechnology Center (University of Illinois at Urbana-Champaign) as described before [35]. Briefly, messenger RNA was isolated from total RNA using the Oligotex mRNA Mini kit (Qiagen, CA). First and second strand cDNA were synthesized from 200 ng of mRNA using the SuperScript^® ^Double-Stranded cDNA Synthesis Kit (Invitrogen, CA) with 100 μM random hexamer primers (Fermentas, USA). Double-stranded cDNA was cleaned up with a QIAquick Minelute PCR purification column (Qiagen, CA). Double-stranded DNA was nebulized with the nebulization kit supplied with the GS Titanium Library Preparation kit (Roche/454 Life Sciences, CT) following their recommendations (30 psi for 1 minute) and cleaned up with a QIAquick PCR minelute column and eluted in 50 ul EB. Nebulized cDNA was blunt-ended (25 μl water, 10 μl 10× T4 DNA Ligase buffer (NEB), 4 μl 10 mM dNTP mix, 5 μl T4 DNA polymerase (3 U/μl) (NEB), 1 μl Klenow polymerase (5 U/μl) (NEB), and 5 μl Polynucleotide kinase (10 U/μl) (NEB) and cleaned up with a Qiaquick PCR minelute column and eluted in 32 ul EB. A dA-overhang was added at 3' end of cDNA by adding the following to the blunt-ended cDNA: 5 μl 10× buffer 2 (NEB), 10 μl 1 mM dATP and 3 μl Klenow exo-minus polymerase (5 U/μl) (NEB). The reaction was incubated at 37°C for 30 minutes and then cleaned up with a QIAquick MiniElute column and eluted in 10 μl EB. The cDNA was adaptored with Titanium adaptors (Roche/454 Life Sciences, CT) by adding 9 μl water, 25 μl 2× Rapid Ligase buffer (Enzymatics, MA) 5 μl (50 μM) Titanium adapter A/B mix and 1 μl T4 DNA Ligase (600 U/μl (Enzymatics, MA) and incubated the ligation reaction at room temperature for 15 minutes. The reaction was cleaned up using a Qiaquick MiniElute column (Qiagen), eluting the cDNA in 20 μl EB. Adaptored cDNA was run on a E-GEL EX 2% agarose (Invitrogen, CA) following the manufacturer instructions and cDNAs in the size range of 400-800 bp were excised from the gel and purified with a Qiagen's Gel Extraction kit and the cDNA was eluted in 30 μl EB. One μl of the gel-purified cDNA was used as template for amplification in 50 μl PCR reactions containing 10 μl 5× Phusion Buffer HF (NEB), 25 μM Adapter A_For primer (5'CCATCTCATCCCTGCGTGTCTCCGACTCAGACGAGTGCGT3'), 25 μM Adapter B_For primer (5'CCTATCCCCTGTGTGCCTTGGCAGTCTCAGT3'), 3% DMSO, 10 mM dNTPs and 1 U Phusion polymerase (Finnzymes/NEB, USA). The PCR conditions were as follows: 98°C for 30 seconds, followed by 15 cycles with 98°C for 10 seconds, 68°C for 30 seconds and 72°C for 30 seconds, with a final extension of 72°C for 5 minute and cleaned up with a Qiaquick minelute PCR column.

### Normalization of cDNA library

The cDNA library was normalized according to the protocol described in the Trimmer Direct Kit (Evrogen, Russia). In brief, 300 ng of cDNA were incubated at 95°C for 5 minutes followed by incubation at 68°C for 4 hours in the hybridization buffer included in the kit (50 mM Hepes, pH7.5 and 0.5 M NaCl). After the incubation, the reaction was treated with ¼ units of duplex specific nuclease (DSN). The normalized cDNA was then amplified from 1 μl of DSN-treated cDNA by PCR reactions (10 cycles) described above and gel purified for the fragment size of 400-800 bp as described above.

### 454/GS-Titanium sequencing

After library construction, the samples were quantified using a Qubit fluorometer (Invitrogen, CA) and average fragment sizes were determined by analyzing 1 μl of the samples on the Bioanalyzer (Agilent, CA) using a DNA 7500 chip. The library was diluted to 1 × 10^6^molecules/μl. emPCR was set up using Titanium emPCR kits (Roche) at 0.25 copies of DNA molecules per bead (8.75 μl of 1 × 10^6 ^molecules/μl into 35 million beads). Processing was done per Roche protocols for the remaining emPCR breaking, enrichment, and PTP loading steps.

### Bioinformatics analysis

The sff sequence files were processed by using tools from Roche [36] and in-house Java scripts. Sequences that contain homopolymers (in which 60% over the entire length of the read is represented by one nucleotide) and that are shorter than 100 bp were filtered out. Modified sequence data were *De novo *assembled using the software SeqMan NGen version 2.0 (trial version) from DNAStar [37] at default parameters (Match size, 40 bp; minimum match percentage, 90%; mismatch penalty, 25; gap penalty, 25). Similarly, a combination assembly was done using both the 454-pyrosequencing ESTs and the pre-existing Sanger sequences downloaded from the rainbow trout gene index [15].

## Authors' contributions

MS designed the project, was responsible for generation of the pyrosequence data, data analysis, and drafted the manuscript; CR was responsible for the Sanger-based data, shared in conception of the project; JW assisted with pyrosequence library preparation; GT developed the Swanson doubled haploid line and provided tissues as a source of RNA for pyrosequence data; JY contributed to overall project design and is the corresponding author for manuscript. All authors read and approved the final manuscript.

## Supplementary Material

Additional file 1 **BLASTx top-hit fish species**. Top-hit from BLASTx search matching sequences from fish species Click here for file
